# GBM Cells Exhibit Susceptibility to Metformin Treatment According to TLR4 Pathway Activation and Metabolic and Antioxidant Status

**DOI:** 10.3390/cancers15030587

**Published:** 2023-01-18

**Authors:** Isabele Fattori Moretti, Antonio Marcondes Lerario, Paula Rodrigues Sola, Janaína Macedo-da-Silva, Mauricio da Silva Baptista, Giuseppe Palmisano, Sueli Mieko Oba-Shinjo, Suely Kazue Nagahashi Marie

**Affiliations:** 1 Laboratory of Molecular and Cellular Biology (LIM 15), Department of Neurology, Faculdade de Medicina FMUSP, Universidade de Sao Paulo, Sao Paulo 01246-903, SP, Brazil; 2 Department of Internal Medicine, Division of Metabolism, Endocrinology and Diabetes, University of Michigan, Ann Arbor, MI 48108, USA; 3 GlycoProteomics Laboratory, Department of Parasitology, ICB, University of Sao Paulo, São Paulo 05508-000, SP, Brazil; 4 Biochemistry Department, Institute of Chemistry, Universidade de Sao Paulo, São Paulo 05508-900, SP, Brazil

**Keywords:** GBM, U87MG, A172, Metformin, LPS, antioxidant, cell cycle arrest, apoptosis

## Abstract

**Simple Summary:**

An analysis of metformin (MET) treatment in combination with temozolomide (TMZ) in two glioblastoma cell lines, U87MG and A172, stimulated with lipopolysaccharide (LPS), a TLR4 agonist was conducted. Both cells presented blunted mitochondrial respiration leading to oxidative stress after MET treatment, and decreased cell viability after MET + TMZ treatment. U87MG cells presented increased apoptosis after MET + LPS + TMZ treatment by increment of ER stress, and downregulation of BLC2. A172, with an upregulated antioxidant background, including *SOD1*, exhibited cell cycle arrest after MET + TMZ treatment. The observed differential response was associated with a distinct metabolic status: glycolytic/plurimetabolic (GPM) subtype in U87MG and mitochondrial (MTC) in A172. TCGA-GBM-RNASeq in silico analysis showed that GPM-GBM cases with an activated TLR4 pathway might respond to MET, but the concomitant *CXCL8*/IL8 upregulation may demand a combination treatment with an IL8 inhibitor. MET combined with an antioxidant inhibitor, such as anti-SOD1, may be indicated for MTC-GBM cases.

**Abstract:**

Glioblastoma (GBM) is an aggressive brain cancer associated with poor overall survival. The metabolic status and tumor microenvironment of GBM cells have been targeted to improve therapeutic strategies. TLR4 is an important innate immune receptor capable of recognizing pathogens and danger-associated molecules. We have previously demonstrated the presence of TLR4 in GBM tumors and the decreased viability of the GBM tumor cell line after lipopolysaccharide (LPS) (TLR4 agonist) stimulation. In the present study, metformin (MET) treatment, used in combination with temozolomide (TMZ) in two GBM cell lines (U87MG and A172) and stimulated with LPS was analyzed. MET is a drug widely used for the treatment of diabetes and has been repurposed for cancer treatment owing to its anti-proliferative and anti-inflammatory actions. The aim of the study was to investigate MET and LPS treatment in two GBM cell lines with different metabolic statuses. MET treatment led to mitochondrial respiration blunting and oxidative stress with superoxide production in both cell lines, more markedly in U87MG cells. Decreased cell viability after MET + TMZ and MET + LPS + TMZ treatment was observed in both cell lines. U87MG cells exhibited apoptosis after MET + LPS + TMZ treatment, promoting increased ER stress, unfolded protein response, and BLC2 downregulation. LPS stimulation of U87MG cells led to upregulation of *SOD2* and genes related to the TLR4 signaling pathway, including *IL1B* and *CXCL8*. A172 cells attained upregulated antioxidant gene expression, particularly *SOD1*, *TXN* and *PRDX1-5*, while MET treatment led to cell-cycle arrest. In silico analysis of the TCGA-GBM-RNASeq dataset indicated that the glycolytic plurimetabolic (GPM)-GBM subtype had a transcriptomic profile which overlapped with U87MG cells, suggesting GBM cases exhibiting this metabolic background with an activated inflammatory TLR4 pathway may respond to MET treatment. For cases with upregulated *CXCL8*, coding for IL8 (a pro-angiogenic factor), combination treatment with an IL8 inhibitor may improve tumor growth control. The A172 cell line corresponded to the mitochondrial (MTC)-GBM subtype, where MET plus an antioxidant inhibitor, such as anti-SOD1, may be indicated as a combinatory therapy.

## 1. Introduction

Glioblastoma (GBM), a WHO grade 4 astrocytoma, is the most aggressive and malignant brain tumor [1], with an overall survival (OS) of 15 months [2], despite the current standard of care treatment consisting of surgical tumor macroresection followed by radiotherapy and chemotherapy with the alkylating agent temozolomide (TMZ) [3]. The limited effectiveness of therapeutic modalities available has been attributed to tumor invasiveness and high tumor heterogeneity [4]. Moreover, metabolic plasticity guarantees tumor fitness, where a blockade of metabolic pathways has been a focus of combination therapy strategies [5].

Metformin (MET), 1,1-dimethylbiguanide hydrochloride, known for its hypoglycemic action and widely used as the first-line medication for the treatment of type 2 diabetes [6], has been repurposed for cancer therapy. Known MET actions include regulation of AMPK pathway activity and mitochondria oxidative stress through inhibition of the oxidative phosphorylation (OXPHOS) complex I [7]. MET can also inhibit hexokinase activity and reduce cell glucose consumption, as well as act on the NFκB canonical pathway decreasing IL8 [8], IL6, and TNF expression [9]. Moreover, recent studies have shown the role of MET in inhibiting NLRP3 inflammasome activation and IL1β production in alveolar macrophages [10]; involving inhibition of NFκB-NLRP3-mediated endothelial cell pyroptosis [11]; and of fatty acid synthase (FASN) with suppression of the proinflammatory response through the FASN/AKT pathway [12]. Additionally, inhibition of tumor growth using MET treatment has been described for several types of cancer, including colon, breast, prostate, pancreatic, lung, endometrial carcinomas, melanoma, and leukemia [13,14,15,16,17,18]. In particular, MET is a promising therapeutic option for brain tumors, given its hydrophilic property and permeability across the blood–brain barrier, as demonstrated in animal models [19,20]. In fact, the effects of MET on GBM cell viability have been studied previously [21,22,23], and several clinical trials of combination treatment with MET for GBM patients have been conducted [24].

We have previously demonstrated activation of the TLR4 signaling pathway in GBM, mainly the mesenchymal subtype, and upregulation of IL1β and DNA repair genes through late activation of NFκB in GBM cells stimulated with lipopolysaccharide (LPS). The LPS-stimulated GBM cells had decreased tumor cell viability with the use of treatment combining DNA repair inhibitor and TMZ, which proved more effective than treatment with TMZ alone [25].

In the present study, MET treatment, used in combination with TMZ in two GBM cell lines (U87MG and A172) and stimulated using LPS, was analyzed. The aims were to analyze the signaling pathways activated by MET, LPS and TMZ treatment used alone and in combination, and to identify predictive markers of treatment response.

## 2. Materials and Methods

### 2.1. Cell Culture

GBM cellular lines U87MG and A172 were acquired from ATCC. Lineages authentication by short tandem repeats analysis was performed using the GenePrint 10 System (Promega, Madison, WI, USA). Cells were maintained in DMEM (Dulbecco’s Modified Eagle’s Medium) (Thermo Fisher Scientific, Waltham, MA, USA) with the addition of 10% fetal bovine serum (FBS) (Cultilab, Campinas, Brazil), streptomycin (100 µg/mL), and penicillin (100 IU/mL) (Thermo Fisher Scientific). Cells were incubated at 37 °C with 5% CO_2_ and were routinely tested for mycoplasma contamination.

### 2.2. Cell Treatment

**Figure 1:**
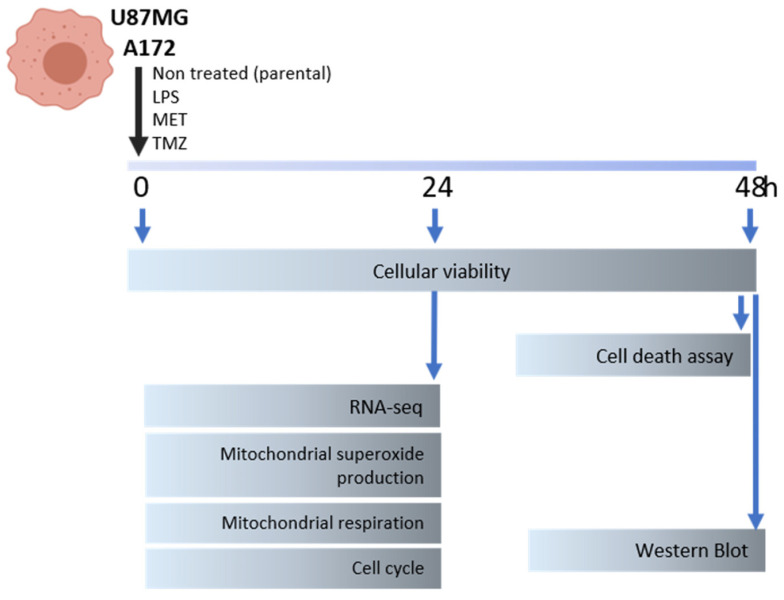
The schematic presentation of the experimental design.

The following reagents: LPS from *Escherichia coli* O55:B5, MET and TMZ (Merck, Readington Township, NJ, USA) were used in U87MG and A172 cell cultures in single or combination treatments. Controls consisted of non-treated cells or treated with DMSO when TMZ was used. Proliferation curves with PrestoBlue reagent (Thermo Fisher Scientific) were performed to determine the half maximal inhibitory concentration (IC50) dose of a single treatment. The IC50 was used for all assays. Assays before (time 0) and after 24 and/or 48 h of treatment were analyzed, according to Figure 1, which shows the schematic experimental design with the time points of the cellular functional analysis: cell viability, apoptosis, cell cycle, mitochondria respiration and superoxide measurements, and transcriptomic analysis.

### 2.3. Cell Viability and Apoptosis Assays

For the cell viability analyses, cells were plated in 96 wells plate (2 × 10^3^ cells/well) and analyzed at different time points (24, 48 h). PrestoBlue Cell Viability Reagent was used according to the manufacturer’s instructions (Thermo Fisher Scientific). Glomax equipment (Promega) was used to evaluate the fluorescence intensity after incubation (excitation at 540 nm, emission at 560 nm). Treatments were done in octuplicate, and two wells without the cell culture medium were used to access the background for each time point to be subtracted from each measurement value.

Cell-death assays of U87MG and A172 cell lines were analyzed after 48h of treatment. Cells were trypsinized, and the medium containing possible necrotic and late apoptotic cells was collected. The Dead Cell Apoptosis Kit (Thermo Fischer Scientific) containing Annexin V conjugated with FITC and propidium iodide (PI) was used following the instructions of the manufacturer. Cell death measurements were performed in the flow cytometry system BD FACSCanto (Beckton Dickinson, East Rutherford, NJ, USA). The analysis was done by FlowJo version 10 (Beckton Dickinson). For the analysis, a non-stained population of cells was used to set the percentage of alive cells. Positivity only for Annexin V was considered as early apoptosis, double positivity for Annexin V and PI was considered as late apoptosis, and positivity only for PI was considered as necrotic cells.

### 2.4. Mitochondrial Superoxide Assay

Production of superoxide by mitochondria after 24 h of treatment in U87MG and A172 cells was assessed by flow cytometry and compared to non-treated cells, in triplicate for each treatment. The MitoSOX Red Mitochondrial Superoxide Indicator kit was used following the instructions of the manufacturer (Thermo Fischer Scientific). MitoSOX fluorescence was assessed in the flow cytometer FACSCanto (Beckton Dickinson). MitoSOX positivity was analyzed by FlowJo version 10.

### 2.5. Mitochondrial Respiration Analysis

The Seahorse XFe24 Analyzer (Agilent Technology, Santa Clara, CA, USA) equipment was used for mitochondrial respiration analysis of U87MG and A172 cell lines after treatment. The Cell Mito Stress Test Kit was used to access mitochondria viability. Cells were plated in the Seahorse plate and treated with MET and LPS single and combined, in triplicate for each treatment, for 24 h, at 37 °C and 5% CO_2_. Next, cells were washed, and the medium was changed to an un-buffered medium and maintained in a 37 °C incubator free of CO_2_. The oxygen consumption rate (OCR) was measured following the Mito Stress program, and treatment was as follow: 2 µM oligomycin, for inhibiting ATP synthase (OXPHOS complex V), and decreasing OCR; 2 µM carbonyl cyanide 3-chlorophenylhydrazone (CCCP), for collapsing the proton gradient, disrupting the mitochondrial membrane, and maximizing OCR through OXPHOS complex IV; 5 µM antimycin A for inhibiting complex III and rotenone for inhibiting complex I, leading to a mitochondria shutdown.

### 2.6. Cell Cycle Analysis

Analyses of U87MG and A172 cell cycle phases were accessed by flow cytometry. Previously to treatment with LPS, MET, and TMZ, cells were synchronized by incubation with FBS-free DMEM with 0.5% bovine serum albumin for 24 h. Subsequently, cells were treated for 24 h in triplicate and fixed with cold ethanol in increasing concentrations (25, 50, 75, 90%). After fixation, cells were washed and incubated with PI. PI fluorescence was accessed by flow cytometry FACSCanto (Beckton Dickinson). Analysis was performed using FlowJo version 10, using the cell cycle interface.

### 2.7. High-Throughput Sequencing for Transcriptome Analysis

Total RNA of U87MG and A172 cells after 24 h of treatment with LPS and/or MET was extracted using the RNeasy mini kit (Qiagen, Hilden, Germany) for the transcriptomic analysis. Untreated cells were considered as the control. Two independent experiments in duplicate were performed for each condition. RNA integrity and concentration were accessed using RNA screentape in the 4200 Tapestation system (Agilent Technologies). The QuantSeq 3’ mRNA-Seq Library Prep kit FWD for Illumina (Lexogen, Vienna, Austria) was used for library construction from 500 ng of total RNA following the recommendations of the manufacturer. The library concentration was measured using the Qubit dsDNA HS Assay Kit (Thermo Fisher Scientific), and the size distribution was determined using the Agilent D1000 ScreenTape System on TapeStation 4200 (Agilent Technologies). Sequencing was performed using the NextSeq 500 platform (Illumina, San Diego, CA, USA) at the next-generation sequencing facility core (SELA) at Faculdade de Medicina da Universidade de São Paulo (FMUSP). Sequencing data were aligned to the GRCh38 version of the human genome and quantified using the R-Bioconductor package QuasR using HiSAT2 as the aligner [26]. The GFF file containing the gene models was obtained from ftp.ensembl.org (accessed on 20 November 2022). Sequencing quality and alignment metrics were assessed with FastQC and RNASEQC, respectively. Downstream analyses were performed in R using specific Bioconductor and CRAN tools, and briefly described. Normalization was performed with edgeR using the trimmed-mean (TMM) method. We used sva to remove occult/unwanted sources of variation from the data. The R-Bioconductor package limma was used to assess differential gene expression in each group, and to perform log2 counts per million reads mapped (CPM) in the transformation of the data. Principal component analysis was performed using the prcomp function from R-stats, and graphically depicted as biplots constructed using ggplot2. To identify modules of co-regulated genes among the differentially expressed genes, we used heatmap and cutree to perform hierarchical clustering and to build heatmaps displaying these modules. We used Pearson correlation as the similarity metric, and the ward D2 clustering algorithm. We used clusterProfiler to perform gene set enrichment analysis for each module of co-regulated genes. Expression data were centered on the mean of each gene. Additional gene set enrichment analyses were performed by online tools such as Gene Ontology [27,28,29] resources and String consortium [30,31]. The metabolic subtype for the cell lines was determined by the analysis of a combined score of marker gene expressions for glycolytic plurimetabolic (GPM) and mitochondrial (MTC subtypes described by Garofano et al. (2021) [32]. We used GSVA [33] to calculate these scores. For the heatmaps, the data were normalized by z-score. The logCPM for each gene was subtracted by the mean and divided by the standard deviation.

### 2.8. Western Blot

Protein extraction of U87MG and A172 cells was performed after 48 h of treatment using the lysis buffer (10 mM Hepes, 1% SDS, 1.5 mM MgCl_2_, 1 mM KCl, 1 mM DTT, and 0.1% NP-40), protease and phosphatase inhibitors (Sigma-Aldrich, St. Louis, MO, USA). Samples were quantified by Qubit protein Assay kit platform (Thermo Fisher Scientific) and solubilized in sample buffer containing 60 mM Tris-HCl, 2% SDS, 10% glycerol, and 0.01% bromophenol blue. A total of 25 µg of proteins were separated by SDS-PAGE and electro-transferred to PVDF membranes, which were directly incubated with blocking buffer (5% bovine serum albumin (BSA) in Tris-buffered saline (TBS) and 0.05% Tween-20 (TBST)) for 1h. Subsequently, samples were incubated with primary antibodies: anti-BCL2 (2876, Cell signaling, Denver, MA, USA) and anti-β-actin (Sigma-Aldrich, A2228, 1:10,000) for loading control, followed by secondary antibody conjugated with horseradish peroxidase for anti-mouse diluted 1:4000 (Abcam, Cambridge, MA, USA) was used for detection of proteins. Immunoreactive bands were detected with the ChemiDoc XRS Imaging System equipment and protein quantification was performed using the ImageJ software (vesion 1.53t).

### 2.9. In Silico Analysis

The astrocytoma dataset from The Cancer Genome Atlas (TCGA) was downloaded from Genomics Data Commons Data Portal [34], and the data were normalized by DEseq software. GBM cases with clinical follow-up data were selected for the analysis. Data analysis was done by heatmap for visualization using z-score to normalize RPKM values.

### 2.10. Statistical Analysis

Statistical analysis was performed using the program SPSS version 23.0 (IBM Corporation, Armonk, NY, USA), Graph Pad Prism (GraphPad Software Inc., San Diego, CA, USA), and R studio [35]. The Kolmogorov–Smirnov test was applied to verify the normal distribution of the results. For non-parametric analysis, Kruskal-Wallis and post hoc Dunn test were used to assess the differences among three or more groups. For two groups comparison, the Mann–Whitney test was used. For parametric analysis, One-way ANOVA and Tukey post hoc test was used, and for multiple variables comparison, two-way ANOVA and Bonferroni or Tukey were used as post hoc tests. Correlation analysis was done by Pearson’s test when parametric, and Spearman’s when non-parametric. The Corrplot package was used for correlation visualization [36]. Statistical significance was considered when *p* < 0.05. The Kaplan–Meier estimator was applied for the TCGA-GPM-GBM subtype using *SOD2* and *CXCL8* expression ratio, where the cases were stratified as high and low according to the mean value for the ratio. Statistical analysis for the survival distribution was performed by Logrank test.

## 3. Results

### 3.1. Characterization of U87MG and A172 GBM Cell Lines

**Figure 2:**
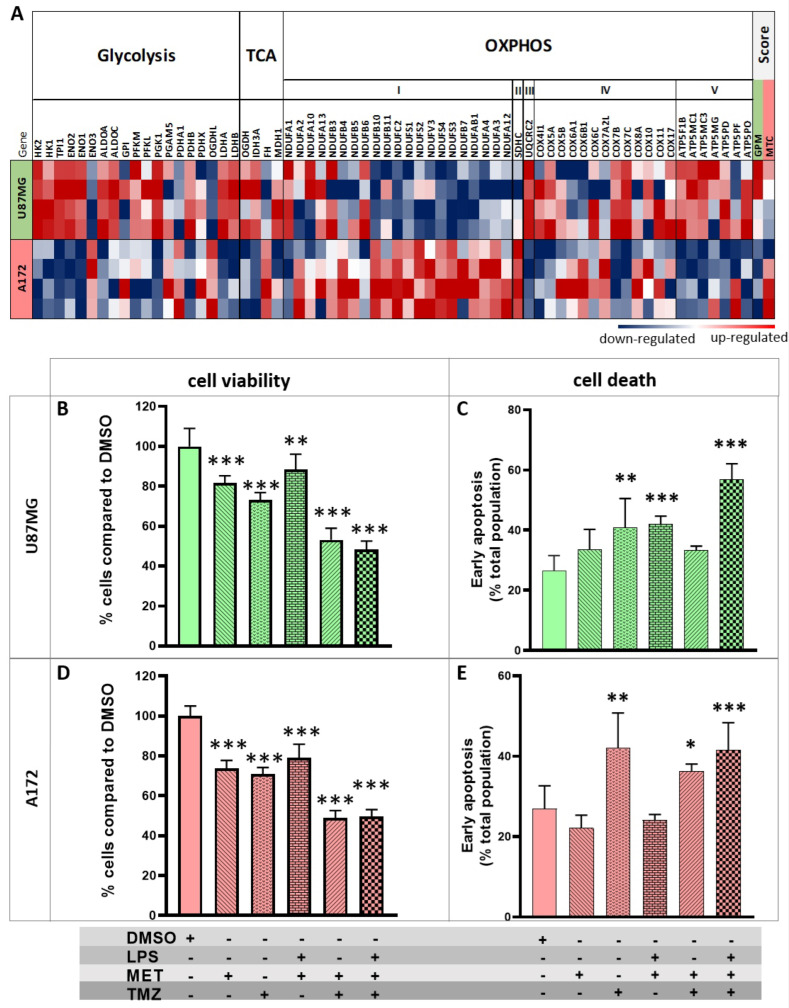
Cell viability and death assays for A172 and U87MG treatment with LPS, MET, and TMZ. (**A**) Heatmap presenting expression values for the genes related to glycolysis, TCA cycle and oxidative phosphorylation normalized by z−score. A score value for the expression of genes attributed as a marker for glycolytic plurimetabolic (GPM) and mitochondrial (MTC) GBM subtypes according to Garofano’s (2021) classification [32]. Upregulated genes are presented in red and downregulated genes in blue. Graph bars representing the viability plotted for the single and combined treatments for LPS, MET, and TMZ in U87MG; (**B**) and A172 (**D**) after 48 h of treatment (**) *p* < 0.01, (***) *p* < 0.001 by one−way ANOVA post hoc Tukey test. Cellular death was analyzed by flow cytometry 48 h after treatments for U87MG; (**C**) and A172; and (**E**), and the results for initial apoptosis are presented. The graphs represent the percentage of the population in initial apoptosis through the positivity for annexin and PI negative in bars for each treatment condition. (*) *p* < 0.05, (**) *p* < 0.01, (***) *p* < 0.001 by two−way ANOVA post hoc Tukey test.

The effect of LPS and MET treatment, used alone and in combination, on U87MG and A172 GBM cell lines, was analyzed given that both present TLR4 expression [37] (Supplementary Figure S1A), and the fact that an increased apoptotic rate with the use of LPS and TMZ co-treatment in U87MG cells has been previously demonstrated by our group [24]. Also, U87MG and A172 cell lines were selected for an additional metabolic intervention with MET because U87MG cells exhibited upregulation of genes related to glycolytic process, while A172 cells showed a marked upregulation of genes related to complex I of OXPHOS, as evidenced by transcriptome analysis (Figure 2A). Moreover, the overall expression levels of genes attributed as markers for the glycolytic plurimetabolic (GPM) GBM subtype and mitochondrial (MTC) GBM subtype, according to Garofano et al. (2021) [32], were upregulated in U87MG and A172 cell lines, respectively (Supplementary Figure S1B).

### 3.2. U87MG and A172 Cell Viability and Cell Death with LPS, MET and TMZ Treatment

Cell viability and cell death assays were performed to analyze U87MG and A172 cell proliferation after use of LPS, MET, and TMZ treatment alone and in combination. In a previous study, we described a decrease in U87MG cell viability following the use of LPS + TMZ treatment [25]. By comparison, MET + TMZ treatment after 48 h in the present study led to a more significant decrease in cell viability (53%) (*p* < 0.001, one-way ANOVA post hoc Tukey test, relative to parental cells treated with DMSO), while MET alone led to a decrease of 19%, TMZ alone 37% or LPS + MET 12% (*p* < 0.05, one-way ANOVA post hoc Tukey test, relative to parental cells with DMSO) (Figure 2B). Analyses were performed at 24 and 48 h, and higher differences for the treatments group was observed after 48 h (Supplementary Figure S2A). The cell death assay revealed a significant increase in initial apoptosis only with the use of the LPS + MET + TMZ treatment combination after 48 h (57%) (*p* < 0.001, two-way ANOVA post hoc Tukey test, relative to parental cells + vehicle DMSO) (Figure 2C and Supplementary Figure S2B).

A172 cells also showed a significant decrease in cell viability (48%, *p* < 0.001, one-way ANOVA post hoc Tukey test, after 48 h) for the MET + TMZ treatment combination (Figure 2D), yielding similar results to the LPS + MET + TMZ treatment (49%). Moreover, no difference in the cell death assay was observed for treatment alone or combined in A172 cells (Figure 2E). Interestingly, treatment with TMZ alone resulted in a 42% increase in initial apoptosis of A172 cells compared to control cells (A172 cells + vehicle DMSO) (Figure 2E). In both cell lines, no differences were observed in late apoptosis (Supplementary Figure S2C).

### 3.3. Altered Signaling Pathways in U87MG and A172 Cells after LPS and MET Treatment Alone and in Combination

The results of the cell viability assay and cell death for the signaling pathways involved were analyzed by high-throughput sequencing of the transcriptome of both cell lines treated with LPS and MET, alone and in combination.

**Figure 3:**
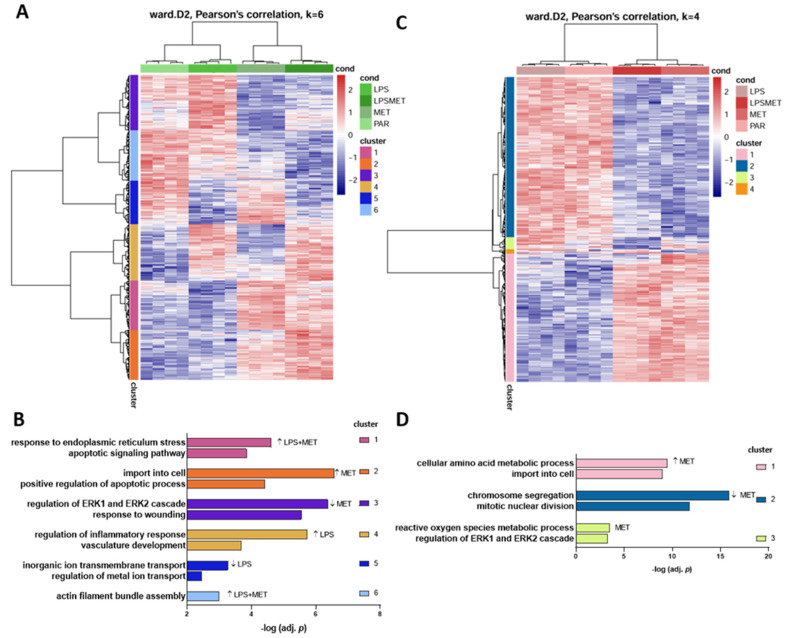
Transcriptome analysis for U87MG and A172 at 24 h after LPS and MET single and combined treatments compared to non−treated cells. A heatmap for the expression values after each treatment is presented and Pearson’s correlation analysis for clusterization of the different groups showed six different clusters for U87MG (**A**) and four clusters for A172 (**C**). The top two gene ontology enrichment pathways identified in each cluster are shown in bars with the −log adj *p* for U87MG (**B**) and A172 (**D**).

The RNASeq of U87MG cells yielded 12,396 genes with 212 differentially expressed genes (DEGs) for LPS treatment, 362 DEGs for MET treatment and 1810 DEGs for combined LPS + MET treatment with an adjusted *p* (adj *p*) < 0.1 compared to non-treated cells. To identify the clusters of DEGs, a Pearson’s correlation analysis was performed, which identified 6 different clusters for the comparison of the four groups (U87MG parental cells, LPS alone, MET alone and LPS + MET treated cells) (Figure 3A). The clusters included enrichment of DEGs associated with different signaling pathways (Figure 3B). Cluster 1 showed upregulated genes after combined treatment (LPS + MET), whereas cluster 2 included upregulated genes only after MET treatment, while both clusters were related to apoptotic signaling pathway on the gene ontology enrichment analysis. Additionally, Cluster 1 included genes related to endoplasmic reticulum (ER) stress response and Cluster 2 to a process of import into cell pathways. Clusters 3 and 6 included genes downregulated after MET treatment, where the genes in Cluster 3 were related to response to wound and regulation of ERK1 and ERK2 cascade pathways, while the genes in Cluster 6 were related to actin filament assembly with increment of downregulation after combined (LPS + MET) treatment. Cluster 4 included upregulated genes after LPS treatment with enrichment for regulation of inflammatory response and vasculature development, and Cluster 5 included downregulated genes after LPS treatment enriched for ion transport and regulation (Figure 3A,B).

The RNASeq of A172 cells detected 13,059 genes, with 1278 DEGs after MET treatment and 1204 DEGs after LPS + MET combined treatment compared to non-treated cells, with an adj *p* < 0.1. Interestingly, LPS stimulation promoted no alteration in DEG profile relative to non-treated cells. The Pearson’s correlation analysis for the DEGs showed four clusters of correlation (Figure 3C). Cluster 1 included upregulated genes after MET treatment with enrichment of genes related to the amino acid metabolic process and import into the cell pathways, while Cluster 2 included downregulated genes after MET treatment related to chromosome segregation and mitotic nuclear division. Cluster 3 also included downregulated genes after MET treatment associated with the reactive oxygen metabolic process. Cluster 4 presented no significant enriched pathway (Figure 3C,D).

### 3.4. U87MG Cells Were Prone to Mitochondrial Stress after MET Treatment

With regard to MET inhibition at the level of complex I of OXPHOS with consequent increase in reactive oxygen species (ROS) production, the MitoSOX assay was performed under the different treatment conditions.

**Figure 4:**
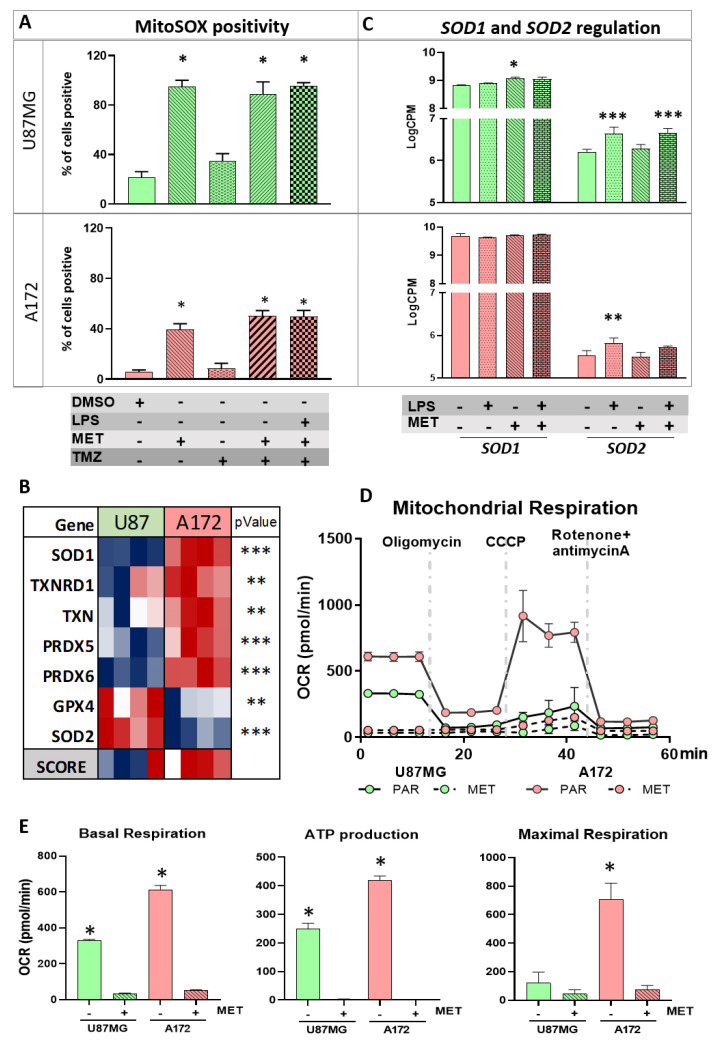
Mitochondrial stress. (**A**) The superoxide production in mitochondria after LPS, MET, and TMZ single and combined treatments for U87MG and A172. Graph bars represent the percentage of positive cells for MitoSOX. (*) *p* < 0.0001, One−wayANOVA post hoc Tukey test; **(B**) heatmap presenting the expression levels of antioxidant-related genes in U87MG and A172 cells. Presenting score values for the pathway for both cells (**) *p* < 0.01, and (***) *p* < 0.001, Limma *t*-test; (**C**) values for logCPM for *SOD1* and *SOD2* represented by the graph bars for U87MG and A172 after LPS, MET and LPS + MET treatment (*) *p* < 0.05, (**) *p* < 0.01, and *p* < 0.001, Limma *t*-test; (**D**) mitochondrial respiration by Seahorse, following the mitochondrial stress analysis. The oxygen consumption rate (OCR) curves along the time interval up to 60 min are presented according to applied drugs; and (**E**) histograms of basal respiration calculated by OCR before oligomycin incubation; ATP production evaluated by oligomycin−OCR subtracted from baseline cellular rate and maximal mitochondria respiration calculated as the value after CCCP−OCR subtracted from the value after rotenone- and antimycin A−OCR for U87MG and A172 in non-treated and MET treated. (*) *p* < 0.0001, one−way ANOVA followed by Tukey test. Red (parental-PAR), blue (MET treated) for U87MG and lilac (PAR), green (MET treated) for A172.

An increase in mitochondrial superoxide production was observed in U87MG cells, as 100% of cells were positive for mitochondrial superoxide after MET treatment, a result replicated for the treatments combining TMZ or LPS (p < 0.0001 compared to non-treated condition, one-way ANOVA, post-hoc Tukey test) (Figure 4A and Supplementary Figure S3A). By contrast, A172 cells showed only 50% positivity when treated with MET, a rate unchanged by the treatment combination with TMZ or LPS (*p* < 0.0001 compared to non-treated cells, one-way ANOVA, post-hoc Tukey test) (Figure 4A and Supplementary Figure S3B).

The antioxidant genes expressed in mitochondria were evaluated in U87MG and A172 parental cells to better understand the observed difference between the two cell lines. Interestingly, significantly higher expression of an important enzyme responsible for converting superoxide in hydrogen peroxide [38] located in the mitochondrial intermembrane (*SOD1*) was observed in A172 cells at higher levels than in U87MG cells (logFC = 0.773 and adj *p* < 0.0001). Additionally, expressions of *TXNRD1*, *TXN*, *PRDX5* and *PRDX6* coding for thioredoxin reductase 1, thioredoxin, peroxiredoxin 5 and peroxiredoxin 6 proteins, respectively, located in mitochondria, were higher in A172 cells. In contrast, U87MG cells exhibited higher expression of SOD2 compared to A172 cells (logFC = 0.738, adj *p* < 0.0001). *SOD2* encodes a mitochondrial protein that binds to the superoxide by products of OXPHOS and converts them into hydrogen peroxide and diatomic oxygen [39] (Figure 4B). *GPX4*, a member of glutathione peroxidase that is active in mitochondria, was the only other upregulated antioxidant gene in U87MG cells. Therefore, the number of upregulated genes coding for antioxidant enzymes located in mitochondria was greater in A172 than in U87MG cells, corroborating the results of the MitoSOX assay with massive production of superoxide in U87MG cells. Interestingly, *SOD1* was upregulated after MET treatment in U87MG cell, while upregulation of *SOD2* was observed with LPS stimulation, but not with MET treatment in the two cell lines (Figure 4C).

The mitochondrial respiration of U87MG and A172 cells was measured by a Seahorse metabolic analyzer (Figure 4D). Basal mitochondrial respiration was calculated based on the reduction of the extracellular OCR through the inhibition of ATP synthase by oligomycin. U87MG cells had lower basal respiration than A172 cells (*p* < 0.0001, one-way ANOVA followed by Tukey test), while MET treatment blunted mitochondrial respiration in both cell lines. U87MG cells also had lower ATP production in comparison to A172 cells, calculated by subtracting oligomycin rate from baseline OCR (*p* < 0.0001, one-way ANOVA followed by Tukey test), and no ATP production was observed after MET treatment because the basal respiration was blocked. The maximal mitochondrial respiration capacity was calculated by collapsing the mitochondrial inner membrane and disrupting the mitochondrial membrane potential with CCCP, and by blocking complexes I and III of OXPHOS with rotenone and antimycin A, respectively. A172 cells exhibited significantly higher maximal mitochondrial capacity compared to U87MG cells (*p* < 0.0001), corroborating the high mitochondrial metabolism observed in A172 cells. These results confirmed the effect of MET treatment in both cell lines (Figure 4E). Treatment with LPS alone, or in combination with MET, produced the same results observed after MET treatment in both cell lines (Supplementary Figure S3C).

### 3.5. A172 Cells Showed G2/M Cell Cycle Arrest after MET Treatment

**Figure 5:**
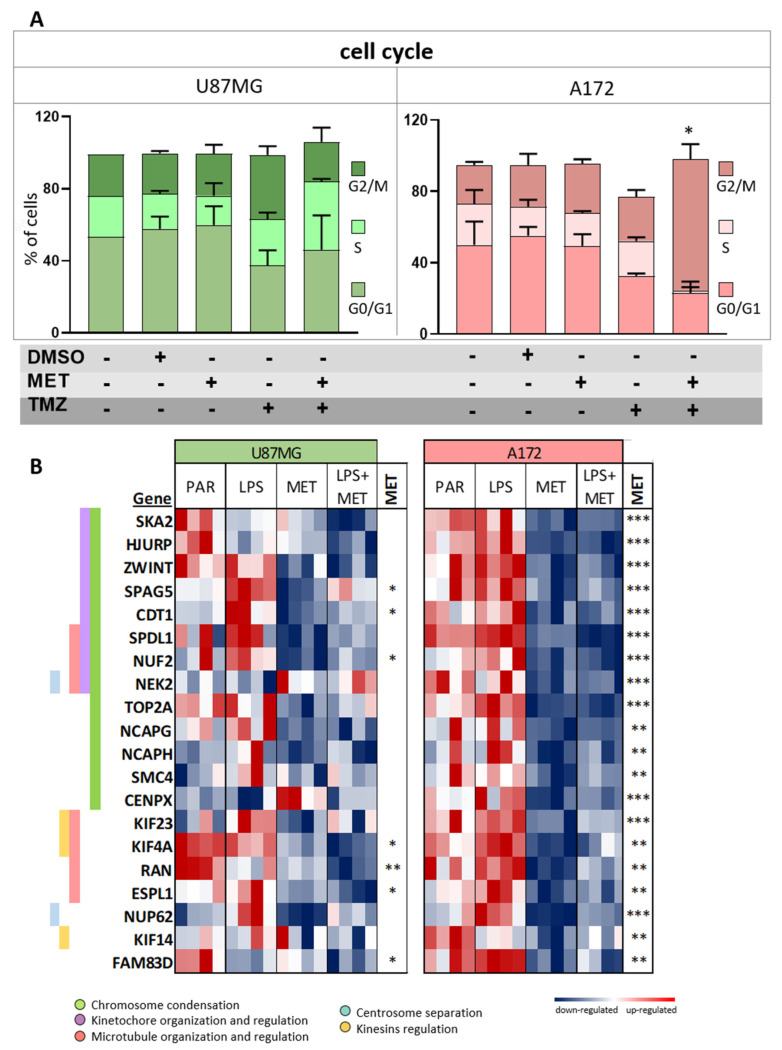
Cell cycle analysis and expression of genes related to chromosome segregation. (**A**) Cell cycle analysis for U87MG and A172 after MET and TMZ single and combined treatments. The bars represent each treatment condition. (*) *p* < 0.0001, two−way ANOVA post hoc Tukey test. G0/G1 phase (bottom bar), S phase (medium bar), G2/M phase (top bar); and (**B**) heatmap of chromosome segregation-related gene expressions after LPS and MET single and combined treatments in U87MG and A172 cells relative to non-treated controls. (*) *p* < 0.05, (**) *p* < 0.01, and (***) *p* < 0.001, Limma *t*-test for MET in comparison to PAR.

Cluster 2 of the RNASeq analysis of A172 cells treated with MET showed a significant downregulation of genes related to chromosome segregation, in congruence with the results of the cell cycle assay. Interestingly, MET treatment alone of A172 cells did not lead to cell-cycle arrest. However, a significant increase in A172 cells (75%) at the G2/M phase, together with a shortened S-phase, was observed with the use of MET + TMZ combined treatment for 24 h (*p* < 0.0001, two-way ANOVA post-hoc Tukey test) (Figure 5A and Supplementary Figure S4A,B). In fact, a significant downregulation of 20 genes associated with chromosome segregation was detected in A172 cells after MET treatment, with a similar result after combined MET + LPS treatment in the RNASeq analysis (Figure 5B). These genes were related to different roles, such as chromosome condensation, kinetochore and microtubule organization and regulation, centromere separation and kinesin regulation. Among these genes, *NUP62*, *SKA2*, *TOP2A*, and *HJURP* were the most downregulated in MET-treated A172 cells compared to non-treated cells (logFC < −0.4, adj *p* < 0.005), while none had significant differential expression in U87MG cells under the same treatment conditions (Figure 5B).

### 3.6. U87MG and A172 Cells Showed Upregulation of ER Stress and U87MG Cells Proved Prone to Apoptosis after MET Treatment

**Figure 6:**
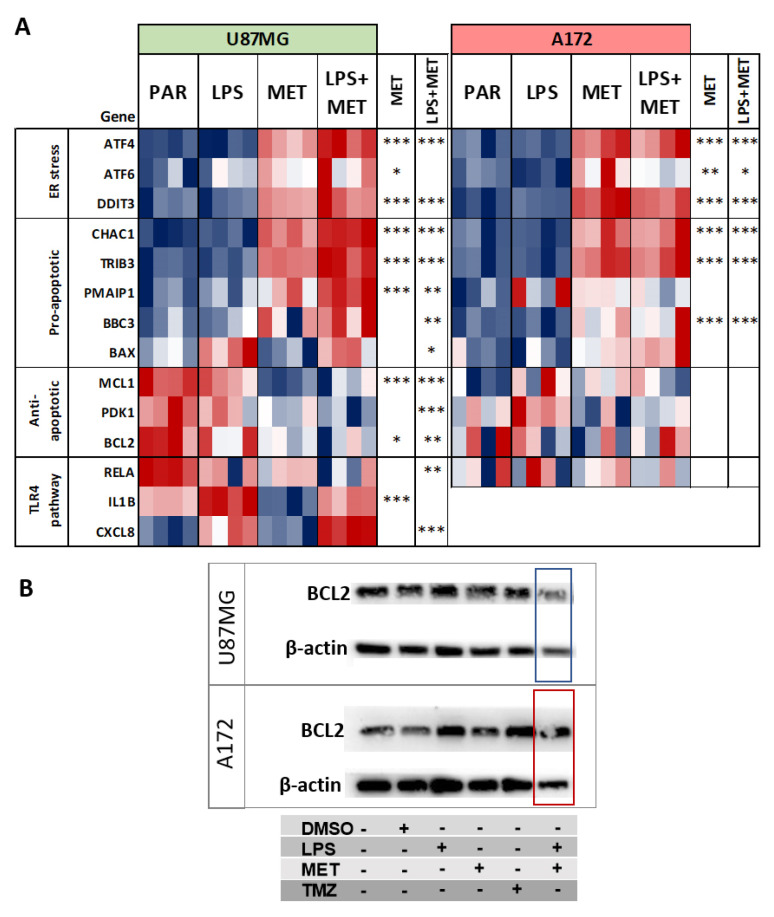
ER stress, pro- and anti-apoptotic and TLR4 pathway related gene expressions. (**A**) Heatmap for expression values of genes related to ER stress, pro−apoptotic, anti−apoptotic and TLR4 pathway in U87MG cells and A172 cells after LPS and MET single and combined treatments, (*) *p* < 0.05, (**) *p* < 0.01, and (***) *p* < 0.001, Limma *t*-test for LPS + MET combined and MET single treatment compared to non−treated cells; and (**B**) Western blot results for BCL2 of U87MG and A172 parental, DMSO, LPS, TMZ, MET single treated cells. β-actin was used for protein loading control.

ER stress-related genes *ATF4*, *ATF6* and *DDIT3* (coding for CHOP), were upregulated in both cell lines after treatment with MET or LPS + MET. Additionally, pro-apoptotic genes *CHAC*, *TRIB3*, *PMAIP1*, *BBC3* and *BAX* were also upregulated in both cell lines, but more significantly in U87MG cells and after combined LPS + MET treatment (3.2 × 10^−11^ < *p* < 0.02, compared to non-treated cells). Additionally, after MET or LPS + MET treatment, U87MG cells exhibited significant downregulation of anti-apoptotic genes, including *MCL1*, *PDK1* and *BCL2* (*p* < 0.005) (Figure 6A). Notably, the decrease in *BCL2* expression was confirmed at the protein level by Western blot in U87MG cells after MET + LPS treatment (Figure 6B, original Western blot is in Supplementary information original images). By contrast, none of these anti-apoptotic genes were downregulated in A172 cells after treatment with MET or LPS + MET.

Another difference between U87MG and A172 cells involved TLR4 signaling pathway activation. Expression of *RELA*, coding for p65 subunit of NFκB, and of *IL1B,* was downregulated in U87MG after MET treatment, most significantly for *IL1B* expression. However, higher upregulation of *CXCL8,* coding for IL8, was observed after LPS + MET treatment compared to LPS treatment alone. By contrast, A172 cells showed no differential expression for *RELA*, while no *CXCL8* and *IL1B* expression was detected for any of the treatment conditions (Figure 6A).

### 3.7. In Silico Validation of the Results in the TCGA-GBM-RNASeq Dataset

**Figure 7:**
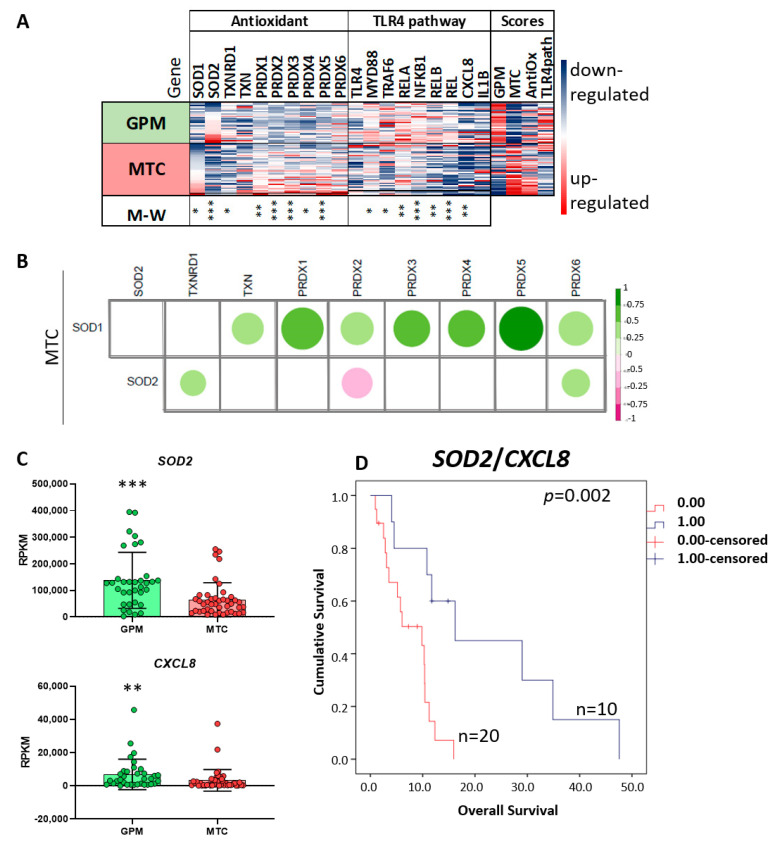
In silico validation of antioxidant and TLR4 pathway-related gene expressions in TCGA GBM-RNASeq dataset. (**A**) Heatmap of antioxidant and TLR4 pathway-related gene expressions normalized by z−score in 34 GPM and 43 MTC GBM subtypes according to Garofano’s classification. The gene signatures for each case were calculated, and a score value was designated and normalized by z−score. (*) *p* < 0.05, (**) *p* < 0.01 (***), *p* < 0.001 (Mann−Whitney test); (**B**) in MTC, Spearman’s correlation analysis showed strong correlation between the expression of *SOD1* and other antioxidant genes. The size of the circles is proportional to the *p* values, and positive (green) and negative (pink) correlations are presented according to the rho values in the bar scale at right. (**C**) RPKM values of *SOD2* and *CXCL8* for GPM− and MTC−GBM subtypes, graph bar presenting the mean values. Each circle represents a GBM case. (**) *p* < 0.01 (***), *p* < 0.001 (Mann−Whitney test); (**D**) in GPM, longer OS was presented by GBM cases with higher *SOD2*/*CXCL8* ratio in a Kaplan−Meier graph, *p* = 0.002 by log rank test (four cases were censored).

Of the 160 GBM cases in the TCGA-RNASeq dataset, 77 were stratified according to the metabolic classification proposed by Garofano et al. (2021) [32] into GPM (*n* = 34) subtype with similarities to the U87MG cell line, and MTC (*n* = 43) subtype with similarities to the A172 cell line. Interestingly, antioxidant genes, including *SOD1*, *TXN* and *PRDX1*-5, were upregulated in MTC cases (Figure 7A and Supplementary Figure S5), whereas *SOD2* (Figure 7A) and *TXNRD1* were upregulated in GPM (*p* < 0.05, Mann–Whitney test). Moreover, expression levels of antioxidant genes were significantly correlated to *SOD1* expression in MTC (*p* < 0.05, Spearman’s test) (Figure 7B). In contrast, genes related to the TLR4 signaling pathway, including *TLR4*, *MYD88*, *TRAF6*, subunits of NFκB (*REL*, *RELA*, *RELB*, *NFKB1*) and *CXCL8*, were upregulated in GPM (*p* < 0.05, Mann-Whitney test) (Figure 7A and Supplementary Figure S5). More specifically, expression of *SOD2* and *CXCL8* was higher in GPM-GBM than in MTC-GBM (Figure 7C) and the analysis of the impact of upregulation of these two genes, according to the Kaplan–Meier estimator, showed that *CXCL8* upregulation was more negative than *SOD2* upregulation, as GPM-GBM cases with lower SOD2/CXCL8 ratio had an OS of 7.72 months compared to 22.26 months for cases with a higher ratio (*p* = 0.002, Logrank test) (Figure 7D). Taken together, this in silico analysis of the MTC-GBM subtype revealing upregulation of antioxidant genes, especially *SOD1*, and the GPM-GBM subtype showing upregulation of *SOD2* and TLR4 pathway-related genes, mirrors the findings observed for A172 and U87MG cells, respectively.

## 4. Discussion

GBM heterogeneity is a major factor limiting the effectiveness of therapeutic strategies available, creating the need to identify biomarkers to better stratify these tumors for specific combination therapies. We analyzed the response of two GBM cell lines to treatment with MET, LPS and TMZ, used alone and in combination. The U87MG cell line harboring the *NF1* mutation and A172 cell line with *RB1* mutation, classified as the mesenchymal GBM subtype with the worst prognosis, were selected to investigate the effects of these treatment conditions. These two specific cell lines were also chosen for their distinct metabolic profile, where U87MG has a GPM profile and A172 a MTC profile, according to Garofano’s proposed GBM stratification based on metabolic pathways [32]. Differences of response to MET were already associated to mutational status of GBM cell lines [40]. Herein, we investigated MET response associated with the metabolic status of GBM cell lines.

Decreased cell viability was detected after MET + TMZ and MET + LPS + TMZ treatments in both cell lines, corroborating previous reports of anti-tumor effects of MET + TMZ [41,42]. Combined LPS + MET treatment has been previously tested in a mouse model of colon rectal cancer, with decreased tumor cell migration and longer OS in this animal model [13]. However, to our knowledge, this is the first study of LPS + MET treatment in gliomas.

A MET effect on mitochondrial respiration was confirmed in U87MG and A172 cells. Although A172 cells exhibited mostly oxidative respiration, with higher expression of genes coding for complex I of OXPHOS compared with U87MG cells, MET treatment reduced ATP production and oxygen consumption in both cell lines. The decoupling of electron transport induced by MET led to oxidative stress with superoxide production, ER stress and unfolded protein response (UPR) activation in both cell lines. Elevated superoxide after MET treatment was previously described in hepatocellular carcinoma [43], and pancreatic cancer cells, where superoxide accumulation in the mitochondrial matrix was associated with alteration of superoxide dismutase (SOD) expression [16]. SODs are antioxidant proteins responsible for converting superoxide radicals into hydrogen peroxide. SOD1 is localized in cytosol and mitochondrial intermembrane space and SOD2 in mitochondrial matrix [39,44]. Interestingly, A172 cells exhibited high expression of SOD1 and of several other antioxidant genes coding for proteins located in the organelle, possibly explaining the lower production of mitochondrial ROS detected after MET treatment. Moreover, A172 cells had upregulated expression of pro-apoptotic genes, with no change in expression of anti-apoptotic genes and, consequently, no increase in apoptosis after MET treatment. Nevertheless, in A172 cells, MET treatment promoted cell-cycle alteration with G2/M arrest due to downregulation of several genes related to chromosome segregation. In particular, genes related to kinetochore (*HJURP*, *ZWINT*), centromere (*NUF2*, *NEK2*, *CENPX*), mitotic spindle (*SPDL1*, *SPAG5*), chromatid separation (*TOP2A*, *NCAPG*), chromosome assembly (*NCAPH*, *SMC4*) and microtubule binding (*CDT1*, *FAM83D*), were downregulated. The kinetochore is built in the centromere and connects the chromosome to microtubules. The NDC80 complex (coded by *NUF2*), the kinetochore structural component, maintains microtubule attachment, and its blockage affects chromosome segregation stability [45]. *CDT1* is associated with the stable attachment of microtubule to kinetochore in the formation of the pre-DNA replication complex [46]. *RAN* [47], *NEK2* [48], *SPDL1* are related to microtubule positioning, where the latter plays this role by recruiting dynein for kinetochore [49]. Kinesins, *KIF4*, *KIF23* and *KIF14*, are important molecules responsible for microtubule transportation and positioning [50], which were also downregulated in A172 cells after MET treatment. Therefore, treatment with MET alone led to cell-cycle arrest, but this intervention proved insufficient to induce cell death of A172 cells. In a bid to identify an analogy of these findings with human GBM cases, the GBM-RNASeq dataset of the TCGA was analyzed. The MTC-GBM subtype, corresponding to the A172 cell line expression profile, showed upregulation of *SOD1* expression and significant correlation with antioxidant gene expressions, predominantly with the *PRDX* family, *PRDX1*-5, and with *TXNRD1*. Given this increased antioxidant state may blunt the apoptotic response, antioxidant inhibitors may represent an alternative combination therapy for the MTC-GBM subtype. Previous studies have demonstrated suppression of the ROS signaling pathway and triggering of apoptosis by a specific SOD1 inhibitor LD100 [51], an efficient copper-chelating agent [52]. Therefore, SOD1 inhibitors, or other antioxidant drugs, may be eligible for use in combination treatment with MET for the MTC-GBM subtype to induce cell-cycle arrest and activation of the apoptotic pathway. Under this condition, SOD1 expression level may be used as an eligibility parameter for this combination therapy.

By contrast, U87MG cells showed upregulation of *SOD2*, with increased expression following LPS stimulation. However, the upregulation of this antioxidant proved insufficient to buffer the massive production of superoxide after MET treatment, exacerbated by the low expression of other mitochondrial antioxidant genes. ER response and UPR activation due to this oxidative stress resulted in increased apoptosis after MET treatment (33%), an increase which was significantly higher with MET + LPS + TMZ treatment (57%). The upregulation of pro-apoptotic genes and downregulation of anti-apoptotic genes, mainly *MCL1*, *PDK1* and *BCL2*, after MET and MET + LPS treatments contributed to the tumor cell death observed. In fact, a previous U87MG in vivo study showed delayed tumor growth with daily MET treatment [40], and better OS with MET + TMZ combined treatment [41]. MET treatment also induced TMZ sensitivity to a resistant GBM cell line [53]. In U251 and T98G GBM cell lines, a decrease of BCL2 and an increase of pro-apoptotic proteins were observed after MET treatment with enhancement of TMZ effect [54].

Additionally, unlike A172 cells, U87MG cells showed activation of the NFκB pathway leading to increased *IL1B* expression after LPS stimulation, confirming our previous evidence [25]. Notably, *IL1B* upregulation persisted after LSP + MET treatment, a phenomenon that might also control tumor growth, as a pyroptotic type of cell death via the cGAS-STING pathway was triggered by persistent stimulation of IL1β [55]. However, LPS treatment also increased *CXCL8*, coding for IL8, a known pro-angiogenic factor in the tumor microenvironment [56], where neovascularization is one of the main characteristics of GBM responsible for its aggressiveness. Moreover, SOD2 upregulation has been associated with poor prognosis in several tumors [57] and was associated with TMZ resistance in GBM cells and in xenograft models [58]. In fact, the transcriptomic analysis of the human GBM cases of the TGCA RNASeq dataset showed the GPM-GBM subtype had upregulation of *SOD2* and genes related to the TLR4 signaling pathway, including *CXCL8*, in comparison to the MTC-GBM subtype. The analysis of the impact of the two pro-tumoral genes, *SOD2* and *CXCL8*, showed a shorter OS for the GPM-GBM cases with lower *SOD2*/*CXCL8*, indicating that increased *CXCL8* expression may be more deleterious. Increased CXCL8 (IL8) expression may be addressed by a neutralizing IL8 monoclonal antibody, which has been tested in a phase I clinical trial for metastatic or unresectable solid tumors and in ongoing studies evaluating its effect in reducing mesenchymal features in tumor cells, rendering them less resistant to treatment [59]. To date, no SOD2 pharmacological inhibitors have been tested. Further in vivo studies are needed to determine the efficacy of the suggested combination therapies for GBM treatment.

## 5. Conclusions

In conclusion, U87MG, a mesenchymal GBM cell line with GPM metabolic background, responded with increased apoptosis after MET + LPS + TMZ treatment via increased ER stress and UPR response and downregulation of BCL2. A172; however, a mesenchymal GBM cell line with an MTC metabolic background, also attained an upregulated antioxidant status and MET treatment led to cell-cycle arrest. The present in vitro findings suggest that the GPM-GBM subtype with activated inflammatory TLR4 pathway may respond to MET treatment and that combination treatment with CXCL8/IL8-inhibitor may improve tumor growth control. The use of MET treatment, in combination with an antioxidant inhibitor such as anti-SOD1, may be an eligible approach for cases with the MTC-GBM subtype. The efficacy of the suggested combination therapies needs to be tested in in vivo studies.

## Data Availability

Not applicable.

## Supplementary Materials

The following supporting information can be downloaded at: https://www.mdpi.com/article/10.3390/cancers15030587/s1. Figure S1:(A) Immunofluorescence analysis exhibiting the presence of TLR4 (in red) in U87MG and A172 cells; (B) Heatmap showing the differential expression in U87MG and A172 cell lines for marker genes for glycolytic plurimetabolic (GPM) and mitochondrial (MTC) according to Garofano’s classification (2021). Figure S2: U87MG and A172 cell death analyzed by flow cytometry. Figure S3: Superoxide production in mitochondria after LPS, MET, and TMZ single and combined treatments for U87MG. Figure S4: Cell cycle analysis for U87MG and A172 after LPS, MET and TMZ single and combined treatments. Figure S5: Heatmap of antioxidant genes, TLR4 pathway-related genes, marker genes for glycolytic plurimetabolic (GPM) and mitochondrial (MTC) subtypes. Original images: Western blot original figure. See Ref. [60].
