# Supplementary material for: GBM Cells Exhibit Susceptibility to Metformin Treatment According to TLR4 Pathway Activation and Metabolic and Antioxidant Status
Source: Cancers (Basel). 2023 Jan 18;15(3):587. doi: 10.3390/cancers15030587 (PMC9913744; doi:10.3390/cancers15030587)
Supplement: Supplementary file 1 [file cancers-15-00587-s001.zip › cancers-2142162-supplementary.pdf]

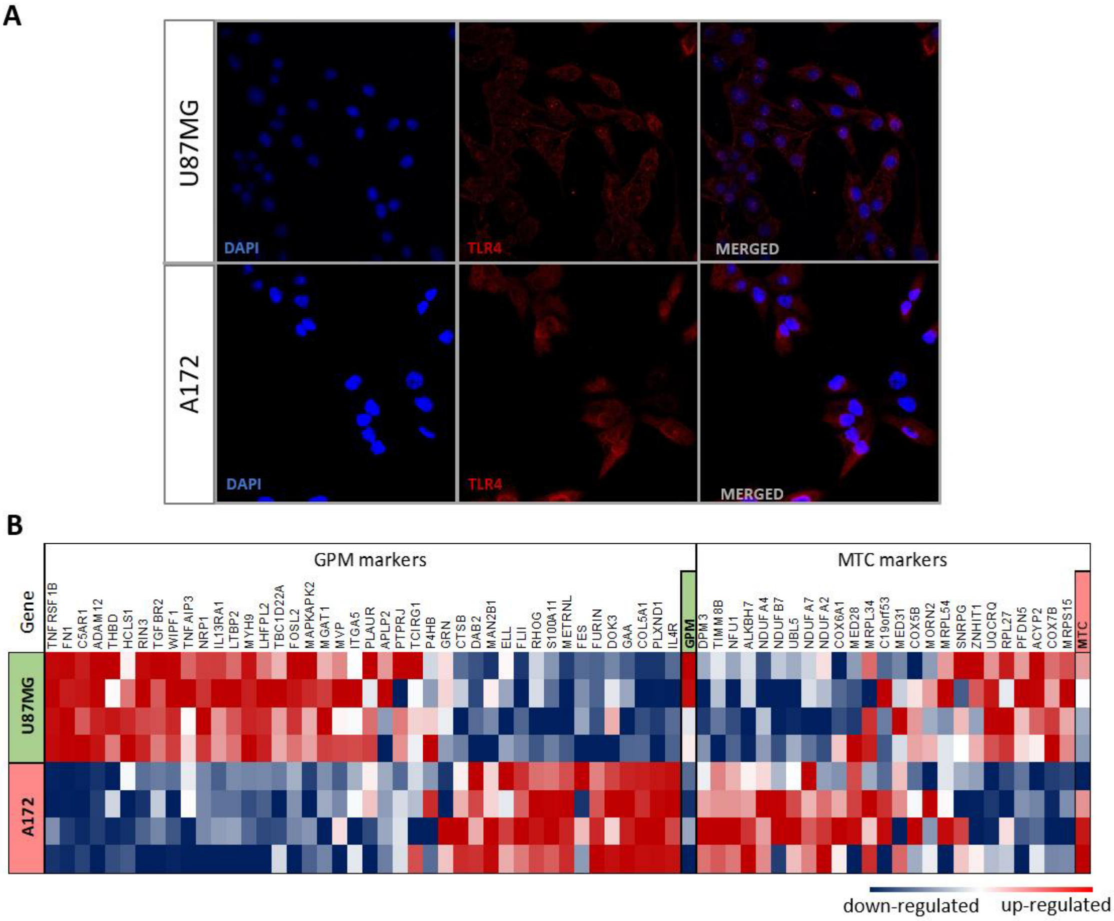

Figure S1. (**A**) Immunofluorescence analysis exhibiting the presence of TLR4 (in red) in U87MG and A172 cells. The nuclei were stained with DAPI. (**B**) Heatmap showing the differential expression in U87MG and A172 cell lines for marker genes for glycolytic plurimetabolic (GPM) and mitochondrial (MTC) according to Garofano's classification (2021) [32] normalized by z-score. Up-regulated genes are presented in red and downregulated genes in blue.

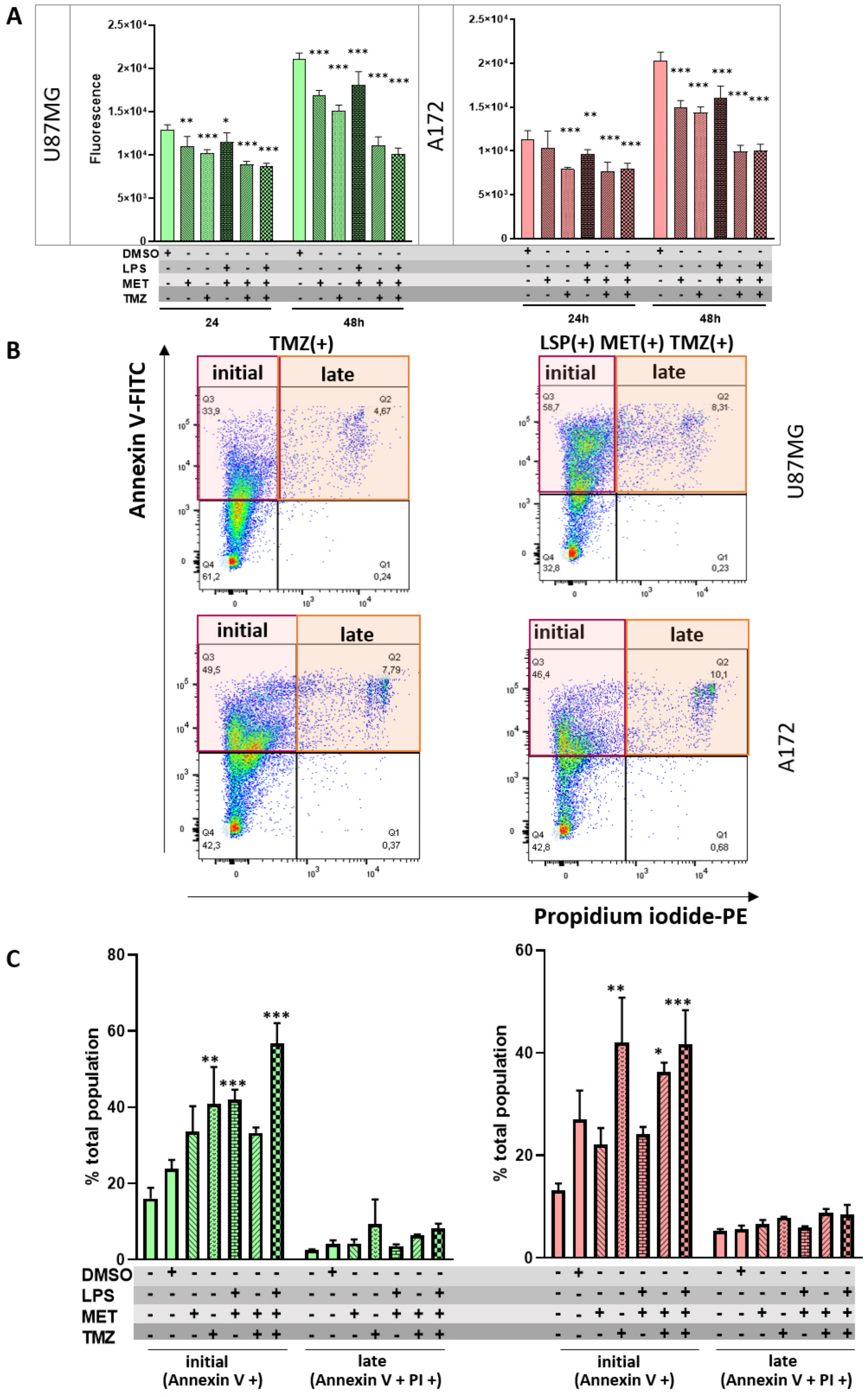

Figure S2. U87MG and A172 cell death analyzed by flow cytometry. (**A**) Graph bars representing the viability plotted for the single and combined treatments for LPS, MET, and TMZ in U87MG and A172 at two different time points (24 and 48 h) (*) $p < 0.05$, (**) $p < 0.01$, (***) p<0.001 (Two-way ANOVA post hoc Tukey test) (**B**) Representation of different cell populations observed in the apoptosis assay of U87MG and A172 cells treated for TMZ alone and LPS+MET+TMZ combination. Initial

and late apoptosis are highlighted in red and orange bars, respectively. (**C**) The values in percentage for initial and late apoptosis are presented in bar-graphs for each treatment condition, where annexin V positive and propidium iodide (PI) negative corresponds to initial apoptosis and annexin V and PI positive to late apoptosis. (*) $p < 0.05$, (**), $p < 0.01$, (***), $p < 0.001$ (Two-way ANOVA post hoc Tukey test).

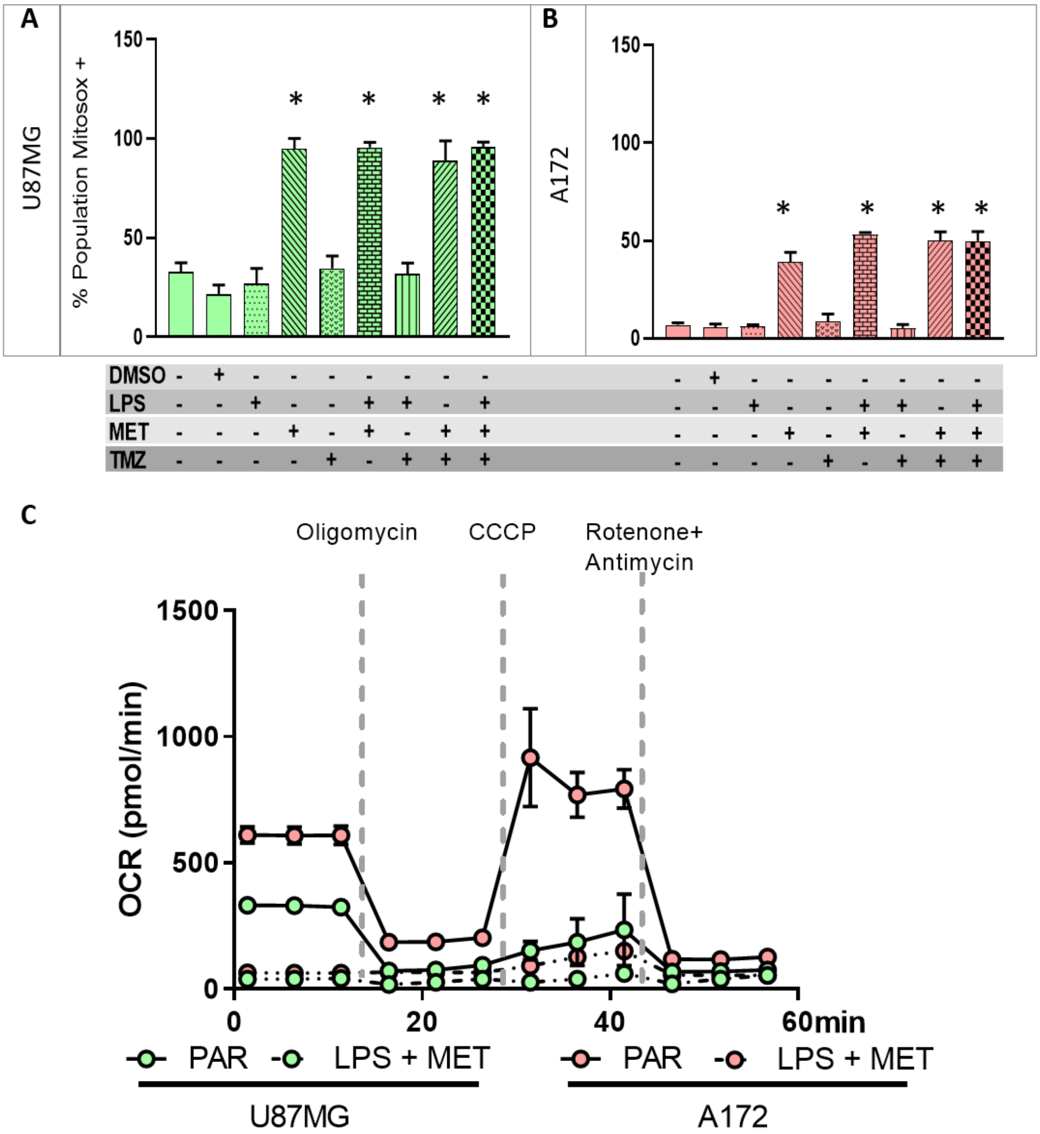

Figure S3. Superoxide production in mitochondria after LPS, MET, and TMZ single and combined treatments for U87MG (**A**) and A172 (**B**). Bar-graphs represent the percentage of positive cells for MitoSOX. (*) $p < 0.0001$, one-way-ANOVA post hoc Tukey test. (**C**) Mitochondrial respiration analysis , following the mitochondrial stress analysis. The curves of the oxygen consumption rate (OCR) along the time interval up to 60 minutes are presented according to drug treatment. The analysis was performed for the LPS and MET single and combined treatments compared to non-treated cells (PAR). CCCP, carbonyl cyanide 3-chlorophenylhydrazone.

A

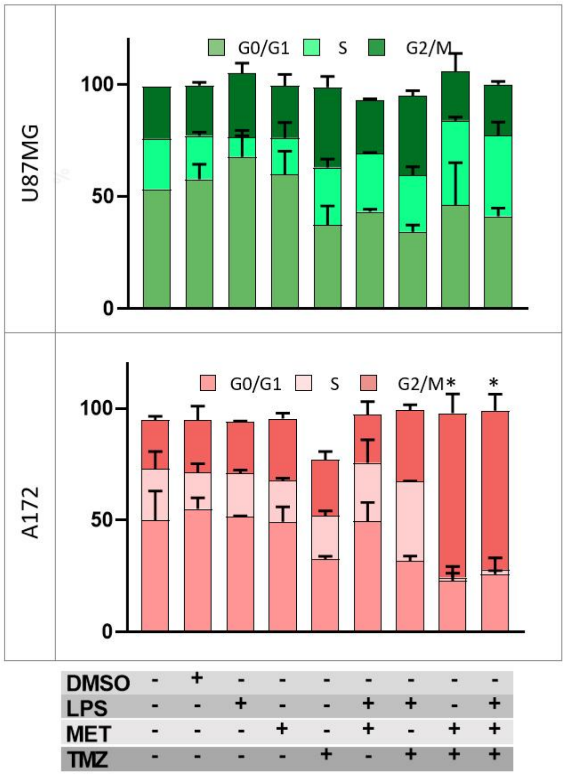

B

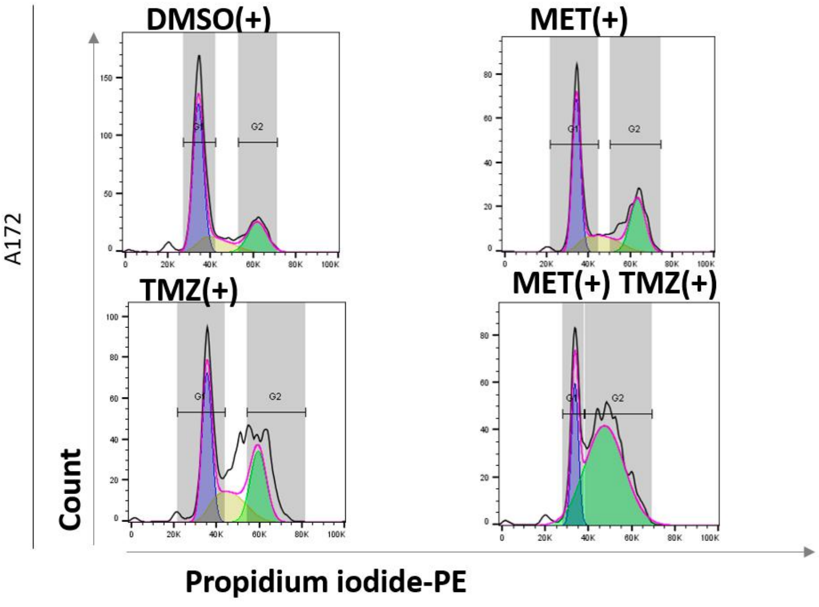

Figure S4. (**A**) Cell cycle analysis for U87MG and A172 after LPS, MET and TMZ single and combined treatments. The bars represent the percentage of cells in the corresponding cell cycle phase in each treatment condition. (*) $p < 0.0001$, two-way-ANOVA post hoc Tukey test. G0/G1 phase (bottom bar), S phase (medium bar), G2/M phase (top bar) (**B**) Cell cycle curves for DMSO, MET and TMZ single and combined treatments of A172 cells. The areas under the curve represent G0/G1 (lilac), S (yellow), and G2/M (green).

A

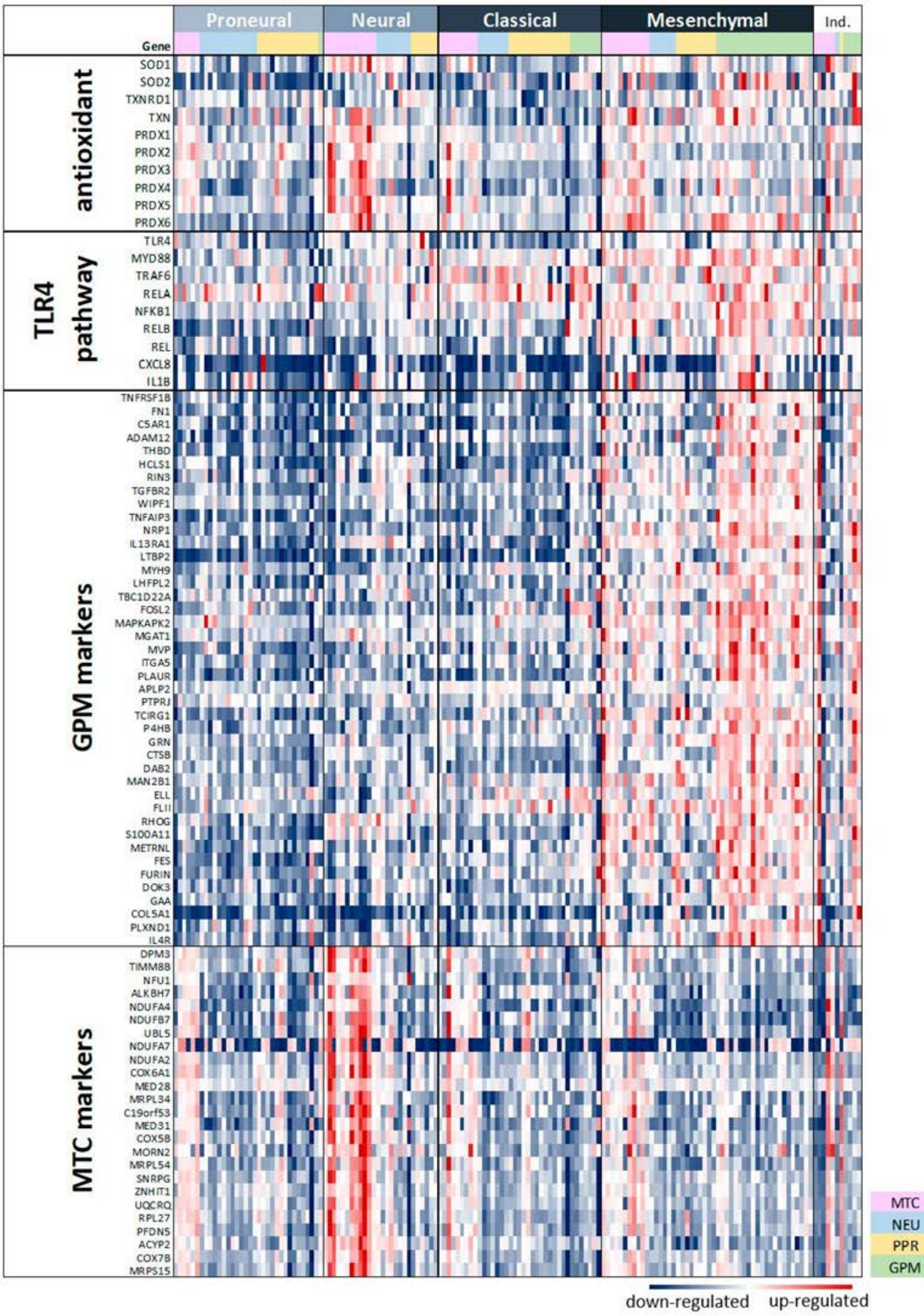

Figure S5. Heatmap of antioxidant genes, TLR4 pathway-related genes, marker genes for glycolytic plurimetabolic (GPM) and mitochondrial (MTC) subtypes according to Garofano´s, 2021 [32] in 160 GBM cases of the TCGA-GBM-RNASeq dataset classified according to Verhaak, 2010 [60] in proneural, neural, classical and mesenchymal molecular subtypes and according to Garofano´s MTC, neural (NEU), proliferative/progenitor (PPR), and GPM metabolic subtypes. The gene expression levels are normalized by z-score, and upregulated genes are in red, and downregulated in blue.

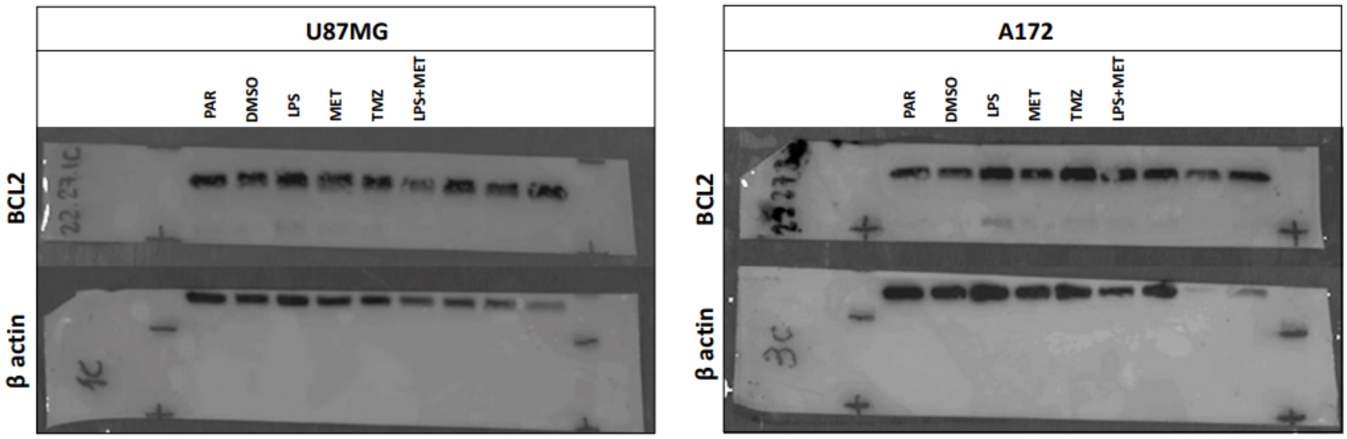

Western Blot images for U87MG and A172 cells. Proteins extracted from each cell line in the designated treatment condition were loaded in one gel.
The membrane was cropped to avoid antibodies cross reaction.
